# Tail Tip Lesions in Mink (*Neovison vison*): Effects of an Additional Hammock in Multilevel Cages

**DOI:** 10.3390/ani8110214

**Published:** 2018-11-19

**Authors:** Celine Kongsli Heimberg, Anna Jespersen, Randi Oppermann Moe

**Affiliations:** 1 Scanvet Animal Health, P.O. Box 3050 Alexander Kiellands Plass, N-0132 Oslo, Norway; 2 Timeline Bioresearch AB, Scheelevägen 2, 22363 Lund, Sweden; annajespersen_dk@yahoo.dk; 3 Faculty of Veterinary Medicine, Norwegian University of Life Sciences, N-0454 Oslo, Norway; randi.moe@nmbu.no

**Keywords:** animal welfare, farmed mink, tail tip lesions, wounds, multilevel cages

## Abstract

**Simple Summary:**

There are several animal welfare concerns in farmed mink, including the occurrence of wounds, such as tail tip lesions. However, little is known about how these lesions develop. An increasing tendency to develop tail tip wounds was reported by Norwegian mink farmers after the introduction of multilevel cages. It appears that the mink jump directly at a presumably high speed from the upper level towards the nest box on ground level, causing the tail to hit the wire mesh several times. This study investigates whether cage design may be involved in the development of tail tip lesions. Specifically, effects of installing an additional hammock in standard multilevel cages, intended to reduce speed during transitions between cage levels and thereby assumed to lower the incidence and severity of tails hitting the wire mesh, were investigated in 600 mink at three farms (300 with hammocks and 300 without hammocks). More tail tip lesions were found in mink housed in cages without a hammock. Further studies are needed to understand the causal relationship between cage design and tail tip lesions in mink, in order to develop recommendations for improved cage designs and thereby improving animal welfare.

**Abstract:**

The occurrence of wounds in different anatomical regions, such as tail tip lesions, is an important welfare concern in farmed mink. This study investigated whether mechanical factors attributed to cage design in multilevel cages may be involved in the etiology of tail tip lesions. Specifically, effects of an additional hammock intended to reduce speed during transitions between cage levels and thereby assumed to lower the incidence and severity of tails hitting the wire mesh were investigated. Three mink farms and a total of 600 mink participated in the study. On each farm, brown female mink (n = 100) were either housed in multilevel cages equipped with plastic hammocks (placed either perpendicular or parallel to the sidewalls) or in standard multilevel cages without hammocks (n = 100). The study was conducted from December to March using singly housed females. Significant differences in the number of tail tip wounds were found between groups with a hammock installed in the cage vs. control groups in two of the farms (*p* = 0.029 and *p* = 0.031), with more wounds developing in cages without a hammock. Furthermore, there was a trend towards difference in the number of tail tip wounds in groups with hammocks installed perpendicular vs. groups with hammocks installed parallel to the cage sidewalls, but a potential farm effect cannot be ruled out. This study is the first to suggest that mechanical factors associated with cage design may play a role in the etiology of tail tip lesions in farmed mink. Further studies are needed to understand the causal relationship between cage design and tail tip lesions in mink.

## 1. Introduction

The welfare of farmed mink is frequently debated. A specific concern is the occurrence of wounds. Wounds on different body parts are considered an indication of reduced welfare due to possible wound-associated pain [1]. The WelFur protocols have been designed as welfare assessment tools for fur animals including mink [2]. An important animal-based measure is to score the occurrence and severity of wounds located in different anatomical regions, i.e., in the ear, scalp, neck, shoulder, thigh, and tail region [2,3].

Regarding wounds in the tail region, they can be found at the tail base and tip. However, the specific anatomical region of the tail is often not reported/differentiated. For instance, one study found 6.7% and 36.9% tail base wounds in male and female mink, respectively, and other tail wounds in 11% and 17.7% of male and female mink, respectively [3]. However, this study did only differentiate between tail base and other tail wounds, not tail tip wounds explicitly. This differentiation is important because the etiology seems to vary between wounds on the tail base, tail tip, and other tail wounds. Furthermore, others reported that 10.7% of pair-housed and 15.2% of group-housed mink had minor, healed tail tip lesions at pelting, whereas 1% of pair-housed and 3.8% of group-housed mink had unhealed lesions [4]. The majority of observations during a post mortem examination at pelting were minor healed wounds or swellings at the tip of the tail [5]. Another report found that 5.4% of the mink had bite wounds on the tip of the tail, and 2% at the tail head at pelting time [6]. For tail base wounds, studies indicate that group composition, social tolerance, and seasonal effects may be important factors [3,6].

However, the etiology of tail tip lesions is not clear. Group-housed mink (in multilevel cages) have more wounds compared to pair-housed mink (in single level cages) [4,6]. However, whether these wounds may be attributed to cage design per se is less clear, since it is difficult to separate effects of cage design, animal density, and social interactions.

As a result of the Norwegian legislation from 2011, most mink are housed in two-levelled wire mesh cages with access to a litter- and straw-filled nest box [7]. The two levels are connected by an opening, and the mink can climb or jump to reach the second level and jump down to reach the ground floor level. Thus, these cages allow for more activity in several dimensions as compared to the previously used one-level cages. This increased possibility for activity, and complexity of the environment is considered beneficial in terms of improving welfare [4].

Interestingly, after the transition into multilevel climbing cage systems, an increased incidence in tail tip lesions in mink has been reported (unpublished). The mink jump more or less directly from the upper level towards the nest box (located on ground level) at a high speed, which causes the tail to hit different parts of the wire mesh (personal observations), suggesting that mechanical factors may be involved in the etiology of tail tip lesions. This problem might be most prevalent in females during the winter when on restricted feeding, where general activity levels increase due to increased pre-feeding pacing and food anticipation as well as stereotypies [8,9,10]. However, effects of adjustments in cage design assumed to reduce speed during transitions between cage levels on tail tip lesions have not been investigated.

In order to gain more knowledge about the etiology of tail tip lesions in mink, this pilot study investigated if an additional hammock installed in standard multilevel cages could reduce the incidence of tail tip wounds in singly housed brown female mink during the winter season. The intention of an additional hammock placed under the opening between the stacked cages was to reduce the speed of the mink, and thereby the incidence and severity of the tail hitting the wire mesh during transitions between cage levels. This is the first study on the occurrence of tail tip lesions in singly housed mink during the winter season.

## 2. Materials and Methods 

**Figure 1:**
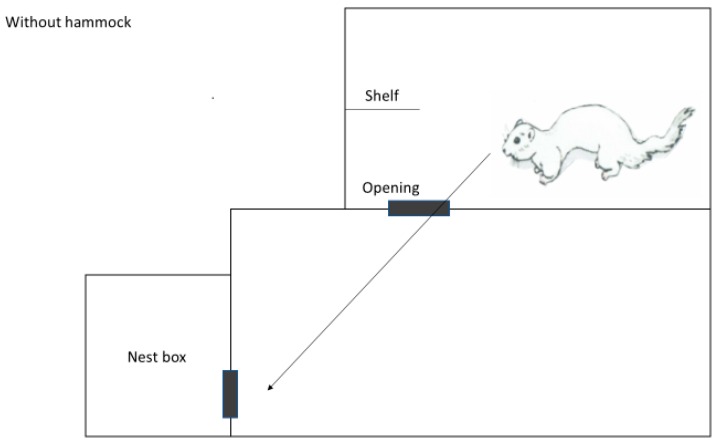
Standard multilevel mink cage (control), view from the left side. The arrow indicates the chosen route of the mink from the upper floor to the nest box.

**Figure 2:**
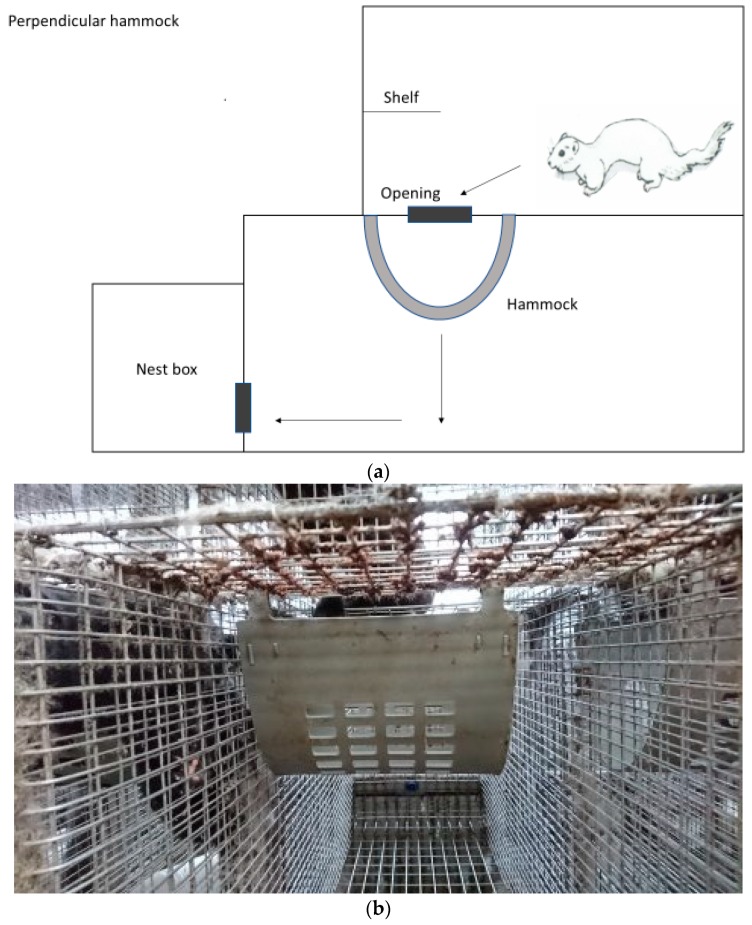
(**a**) Multilevel cage with a hammock installed perpendicular to the sidewalls, view from the left side. The arrows indicate the chosen route of the mink from the upper floor to the nest box. (**b**) Front view of a hammock placed perpendicular to the sidewalls.

**Figure 3:**
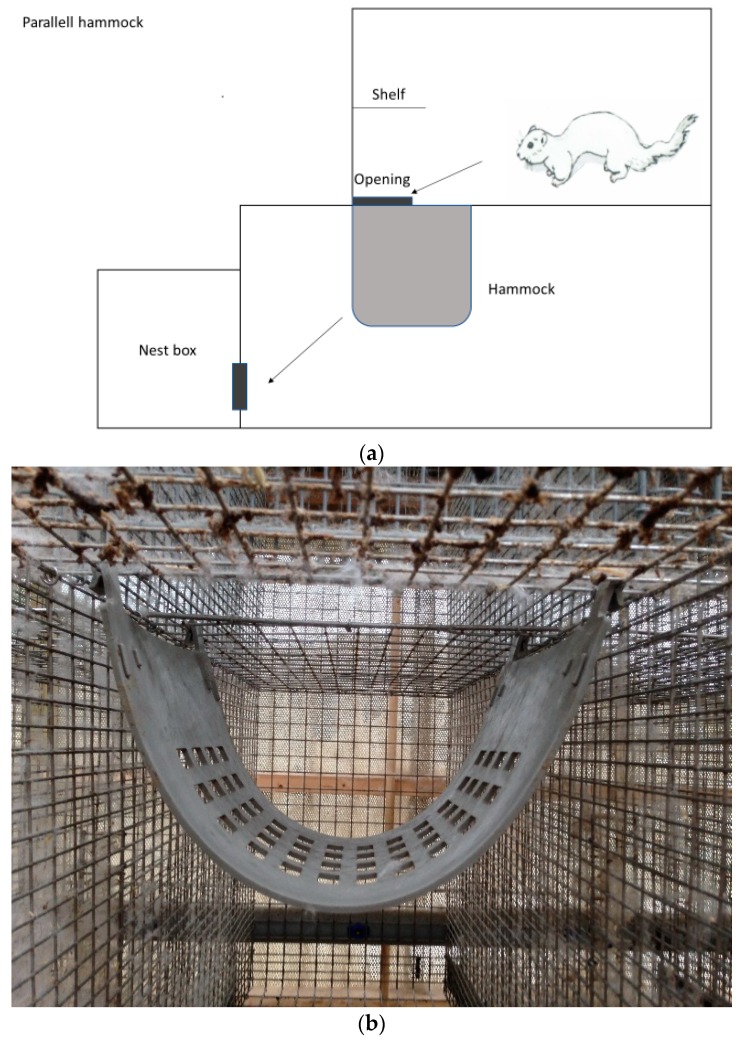
(**a**) Multilevel cage with a hammock installed parallel to the sidewalls, view from the left side. The arrows indicate the chosen route of the mink from the upper floor to the nest box. (**b**) Front view of a hammock placed parallel to the sidewalls.

Three farms were selected based on willingness to participate in the study and their location within the same geographic region in the county of Rogaland in the southwestern part of Norway. The multilevel cages used were of standard size and conformation, consisting of a standard wire mesh cage (L: 0.90 m × W: 0.30 m × H: 0.45 m) with a nest box, connected to a wire mesh top cage (L: 0.70 m × W: 0.30 m × H: 0.45 m). The opening between the standard cage and the top cage was 0.20 m × 0.30 m. The standard cage and the floor of the top cage were made of stainless steel. They contained a wire mesh shelf in front of the upper level and a plastic tube (GVA-group, Ø120 mm × L: 155 mm × thickness: 5 mm) as enrichment, as well as an unlimited supply of straw on top of the nest box. The nest box contained wooden shavings in addition to straw. All mink were fed once daily with fresh conventional wet feed from the local fur animal feed factory. The amount of food given was according to individual body conditioning scoring. Water supply was ad libitum provided from a drinking nipple situated in the back of the bottom cage. Only natural light and headlamps during feeding were used on the farms. At each of the three farms, a convenient and manageable sample size of 200 solitary housed female brown mink was selected to participate in the study (December 2016–March 2017). They were mostly first, and some second year breeders born and raised in social groups in the same kind of cages used in this study. Controls (n = 100) were housed in standard multilevel cages (Figure 1). Another 100 females were housed in standard multilevel cages where plastic hammocks (GVA group, type 04300, Aabybro, Denmark) had been installed. The selection of mink for each group was done randomly by convenience. Two farms managed to install the hammocks perpendicular to the sidewalls (Figure 2a,b) to completely block direct access to the nest box from the upper level. Due to practical reasons (conformation of the wire mesh of the roof of the bottom cage used for fixation of the hammocks), the third farm had to install the hammocks parallel to the sidewalls (Figure 3a,b). Farm 3 is also known to have a higher incidence of tail tip wounds than Farms 1 and 2. None of the mink had tail tip lesions or hair loss of the tail tip at the beginning of this study. This was checked by the first author by visual control before this trial started. Potential impact of climatic interactions were avoided as the recruited farms were based in the same geographical region, and the cages were placed on middle rows in a multi-row barn with open sides to avoid differences in weather impact. The mink in the two groups were gathered in whole middle rows opposite each other in the same barn, with or without hammocks, respectively. Blinding was not possible because it was obvious whether the cage contained an additional hammock or not. All rows were close to each other to avoid differences in microclimate and light. All observations were carried out by the farmers themselves after thorough training by an experienced veterinarian in detecting and assessing tail tip wounds according to the WelFur protocol [2]. Here, tail tip wounds were scored as no visible wounds (score 0), wounds with a diameter <10 mm (score 1), or wounds with a diameter <30 mm (score 2). Mink with wounds >30 mm were to be euthanized and therefore eliminated from this study due to welfare reasons. The visual inspection of mink tails was carried out by the farmer walking behind the cages during feeding once daily. No palpation was performed, but the farmers were instructed to catch mink with wounds to have a closer look. This method of detecting tail wounds was established during the last three years of herd health visits, and was therefore well known to the farmers. No mink in either of the groups had bitten off or lost parts of their tail during this study.

A chi-square test was used to test for independence between the farm and proportion of wounds. Fisher’s exact test was used to test for differences in occurrence of tail tip lesions between groups with and without a hammock. The analyses were carried out in SPSS (IBM Corp. IBM SPSS Statistics for MAC, Version 25. IBM Corp, Armonk, NY, USA).

## 3. Results

The results are presented in Table 1. Because of the limited sample size and Farm 3 having an overall higher incidence of tail tip wounds (*p* < 0.00005), and also installing the hammock differently than Farms 1 and 2, the statistics have been simplified to only contain the parameters with or without hammock, and with or without wounds.

There was a significant difference in the number of tail tip lesions between groups with a hammock installed in the cage vs. control groups in Farms 1 and 3, with more lesions developing in cages without a hammock. There was a trend towards difference between hammock and control group in Farm 2, although this was not significant. No lesions developed in mink from cages with hammocks placed perpendicular to the sidewalls (Farms 1 and 2). Farm 3 with hammocks installed parallel to the sidewalls had an overall higher number of tail tip lesions than Farms 1 and 2.

## 4. Discussion

Briefly, this study found beneficial effects of an additional hammock installed in the standard multilevel cages that reduced the incidence of tail tip lesions in singly housed brown female mink during the winter season. The results may indicate that mechanical factors associated with cage design play a role in the etiology of the development of tail tip lesions in mink. Furthermore, there is possibly a greater effect when positioning the hammock perpendicular rather than parallel to the sidewalls of the cage. This is plausible because mink with a perpendicularly placed hammock need to jump from the top floor onto the hammock, turn 90 degrees, and then continue onto the bottom floor before turning 90 degrees towards the nest box (Figure 2a,b), whereas with a hammock placed parallel to the sidewalls it only works as a stop-over on the way to the nest box (Figure 3a,b). Nevertheless, this theory needs further assessment with a larger number of farms, or installing the hammock parallel versus perpendicular in the same farm to rule out a potential farm effect. The intention of the installed hammock was to reduce the speed of the mink during their transition between cage levels, in order to avoid lesions inflicted by the tails hitting the wire mesh at a presumably high speed. The beneficial effect of hammocks may indicate that they actually contributed to reduce the mink speed during transitions between cage levels, and that the reduced speed resulted in less tail tip wounds because the incidence and severity of tails hitting the wire mesh was reduced. However, it has to be emphasized that no systematic behavioral observations, including positioning in the cage, jumping speed, or frequency were performed. Therefore, further behavioral studies including observation of general locomotor activity, specific behavioral patterns, and speed measurements need to be undertaken.

All cages used were multilevel cages enriched with a nest box with wooden shavings and straw, a wire mesh shelf on the upper floor, and a plastic tube. Environmental enrichments are known to reduce the frequency of stereotypies and fur chewing [8,11] in mink. Studies also found that adding wire shelves in mink cages reduce general locomotor activity [8,10]. Thus, the observed effects could also be attributed to the fact that the hammocks were perceived as an additional shelf-like/hideout-like enrichment, which may have reduced the general activity levels and thereby resulting in less tail tip wounds in mink with access to hammocks. Further studies focusing on general activity, and the potential use of hammocks as additional resting areas need to be investigated.

Mink tend to increase their general activity levels before feeding time, including stereotypies such as pre-feeding pacing [8,9,10,11,12]. Pre-feeding pacing could easily extend to jumping between cage levels, increasing the likelihood of tail injuries if such injuries are related to activity levels. Animals in the present study did not experience intensive, long-term slimming, which is known to cause a significant increase in stereotypies in farmed mink [8,11]. Further studies comparing the occurrence and severity of tail tip lesions in high and low stereotyping lines of mink [13] could reveal whether stereotypies and/or pre-feeding anticipatory behavior may contribute to the development of tail tip lesions.

**Figure 4:**
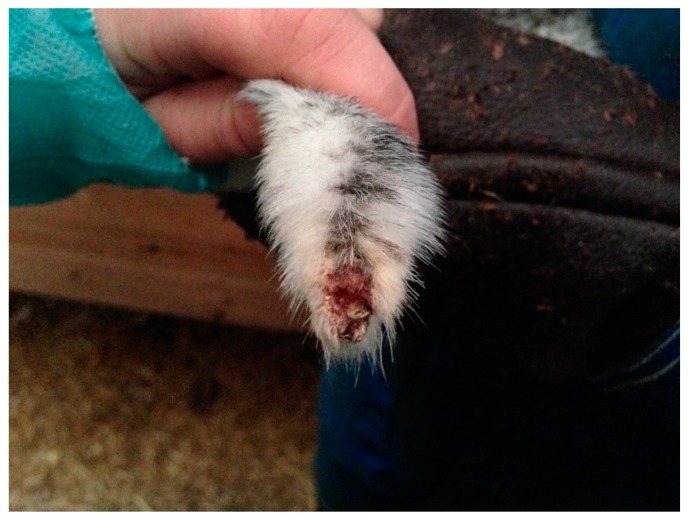
Typical morphology of a wound probably caused by tail hitting the wire mesh, located on the ventral side of the mink tail.

Furthermore, fur chewing could also be suggested to increase the prevalence and severity of tail tip lesions. Fur chewing is characterized by sucking and gnawing [13], and usually starts on the tip of the tail. This exposes, and may weaken the skin, and could in turn predispose the tail tip to injuries. However, neither tail suckling nor the severity of tail tip lesions were observed here, and we can therefore not conclude that tail suckling was an important factor in the occurrence and potential severity of tail tip lesions, or that tail tip lesions were deteriorated due to suckling or due to mechanical factors. Tail tip lesions caused by the mink thwacking their tails against the wire mesh, are typically located on the ventral side of the tail (Figure 4). Small wounds just on the outer 2 mm of the tail tip may just as well be caused by tail suckling/biting, and wounds with extensive crust formation are difficult to assess the etiology and morphology of by visual inspection. The potential links between tail tip lesions and tail suckling warrant further investigations. Although none of the mink participating in this study were missing parts of their tails, this could have been a possible scenario. If so, it would have been difficult to know if tail biting was the primer cause or if the mink had bitten their tails because they were irritated by a wound caused by something else. Animals not leaving wounds to heal is a common problem in veterinary medicine, especially in carnivores and omnivores.

Tail tip lesions could cause an increased risk of infection, which is a well known problem in, e.g., tail-biting in pigs [14]. Indeed, a case report from mink farms in Ontario [15] suggests a connection between tail tip lesions and other wounds as a likely gateway for severe discospondylitis in mink. Interestingly, many of the mink who died of pneumonia (one of the most common causes of mortality in necropsied mink (Aleutean disease free) in Norway during winter time) had small tail tip lesions (personal communication, Veterinary Institute, Sandnes, Norway). Further studies would be needed to understand the relationship between tail tip lesions and general health and mortality in mink.

## 5. Conclusions

Adding an additional hammock in multilevel cages reduced the incidence of tail tip lesions in singly housed female mink during the winter period. This study is the first to suggest that mechanical factors associated with cage design may play a role in the etiology of tail tip lesions in farmed mink. Further studies including behavioral observations, as well as increasing sample size, including farms from more geographical regions, and other hammock designs/placements or other modifications on cage design are needed to fully understand the causal relationship between cage design and tail tip wounds in mink.

## Figures and Tables

**Table 1:** Number of developed tail tip lesions per group on Farms 1–3. The scoring is according to the WelFur protocol [2].

WelFur Wound Scoring	Group	0	1	*p*-Value
Farm 1	With hammock	100	0	0.029
	Control group	94	6	
Farm 2	With hammock	100	0	0.059
	Control group	95	5	
Farm 3	With hammock *	93	7	0.031
	Control group	82	12 **	

* Hammock installed parallel to sidewalls, not perpendicular as Farms 1 and 2; ** Farm 3 had six mink with score two in the control group.
